# A TLR7 antagonist restricts interferon-dependent and -independent immunopathology in a mouse model of severe influenza

**DOI:** 10.1084/jem.20201631

**Published:** 2021-09-02

**Authors:** Julie C.F. Rappe, Katja Finsterbusch, Stefania Crotta, Matthias Mack, Simon L. Priestnall, Andreas Wack

**Affiliations:** 1 Immunoregulation Laboratory, Francis Crick Institute, London, UK; 2 Department of Nephrology, University Hospital Regensburg, Regensburg, Germany; 3 Department of Pathobiology and Population Sciences, The Royal Veterinary College, Hatfield, UK; 4 Experimental Histopathology Science Technology Platform, The Francis Crick Institute, London, UK

## Abstract

Cytokine-mediated immune-cell recruitment and inflammation contribute to protection in respiratory virus infection. However, uncontrolled inflammation and the “cytokine storm” are hallmarks of immunopathology in severe infection. Cytokine storm is a broad term for a phenomenon with diverse characteristics and drivers, depending on host genetics, age, and other factors. Taking advantage of the differential use of virus-sensing systems by different cell types, we test the hypothesis that specifically blocking TLR7-dependent, immune cell–produced cytokines reduces influenza-related immunopathology. In a mouse model of severe influenza characterized by a type I interferon (IFN-I)–driven cytokine storm, TLR7 antagonist treatment leaves epithelial antiviral responses unaltered but acts through pDCs and monocytes to reduce IFN-I and other cytokines in the lung, thus ameliorating inflammation and severity. Moreover, even in the absence of IFN-I signaling, TLR7 antagonism reduces inflammation and mortality driven by monocyte-produced chemoattractants and neutrophil recruitment into the infected lung. Hence, TLR7 antagonism reduces diverse types of cytokine storm in severe influenza.

## Teaser

Distinct types of cytokine storms aggravate respiratory viral infection. This study shows that in severe influenza, TLR7 antagonist treatment ameliorates both type I interferon–driven and interferon-independent immunopathology by blocking TLR7-dependent cytokine production by immune cells, without affecting RIG-I–dependent responses in lung epithelia.

## Graphical Abstract

**Figure:**
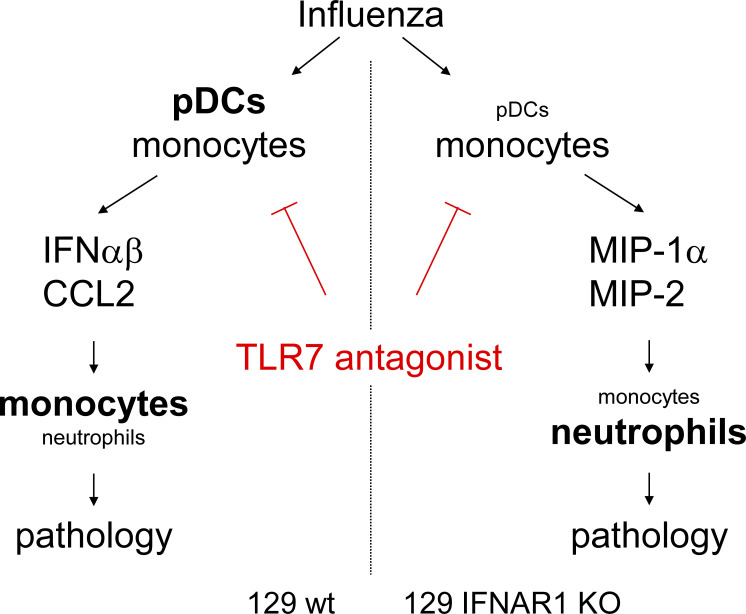

## Introduction

The first line of defense against respiratory viral infection is the anatomic barrier of lung epithelial cells and resident innate immune cells. These cells sense virus-associated molecular patterns by different pattern recognition receptors that cause the induction of both intracellular antiviral defenses and inflammatory mediators such as cytokines and chemokines. These recruit and activate effector cells, contributing to the eventual elimination of the invading pathogen (Iwasaki et al., 2017). However, in some cases, this inflammatory process can grow out of control, leading to excessive cytokine release, called a “cytokine storm” (CS; Liu et al., 2016; Mangalmurti and Hunter, 2020). This hyperinflammation is associated with an increased risk of mortality during viral respiratory diseases (Beigel et al., 2005; Cameron et al., 2008; de Jong et al., 2006; Tisoncik et al., 2012), including COVID-19 (Yang et al., 2020).

The CS takes different forms, involving different cytokine drivers and effector cells, but which mechanisms underlie different types of CS is unclear. Different viruses and virus strains, host genetic background, and physiological factors such as age may influence the specific type of CS triggered. For instance, strong IFN responses and CS have been described for primates infected with a highly pathogenic influenza virus (Baskin et al., 2009), but SARS-CoV-2 is reported to induce only moderate IFN responses and yet causes CS (Hadjadj et al., 2020). Similarly, the elderly are susceptible to severe influenza associated with immunopathology and show weakened IFN and increased IL-6 responses compared with young controls (Pillai et al., 2016). It is therefore clinically relevant to identify different types of CS and understand how they are regulated.

Uncontrolled IFN-I responses are commonly correlated with CS, and elevated IFN-I levels are associated with the most severe cases of severe acute respiratory syndrome (Cameron et al., 2008; Cameron et al., 2007; Channappanavar and Perlman, 2017), Middle East respiratory syndrome (Kim et al., 2016; Min et al., 2016), and influenza (Baskin et al., 2009; Fiore-Gartland et al., 2017). While exogenous administration of IFN-I before infection is protective, IFN-I treatment after the onset of infection increases pulmonary proinflammatory cytokine secretion, innate cell recruitment, and epithelial cell death (Channappanavar et al., 2016; Davidson et al., 2016). Several murine models of antibody-mediated IFN-I receptor (IFNAR) blockade or genetic ablation of IFN-I signaling (IFNAR1 KO) demonstrated the central pathogenic role played by IFN-I during viral infections (Davidson et al., 2014; Li et al., 2017; Major et al., 2020; Teijaro et al., 2013; Totura et al., 2015; Wilson et al., 2013). Of note, viral burden does not always correlate with disease severity. It may therefore be beneficial to reduce or suppress IFN activity in the late phase of viral infection and in situations of uncontrolled inflammation.

To preserve the direct antiviral effects of epithelial-derived IFNs but blunt the damage from immune cell–produced IFN, strategies more refined than antibody-mediated IFN-I blockade are required. Lung epithelia and some immune cells employ RIG-I and TLR3 to produce IFN-I in response to influenza virus infection (Crotta et al., 2013; Ioannidis et al., 2013; Le Goffic et al., 2007). In contrast, plasmacytoid dendritic cells (pDCs), known as professional IFN-I–producing cells (Cervantes-Barragan et al., 2012; Jewell et al., 2007; Li et al., 2014; Siegal et al., 1999; Smit et al., 2006; Swiecki et al., 2010; Takagi et al., 2011), and monocytes recognize influenza virus by an endosomal receptor, TLR7 (and/or TLR8 in humans), to produce copious amounts of IFN-I (Diebold et al., 2004; Lund et al., 2004; Zhang et al., 2016). Therefore, antagonizing TLR7-dependent IFN-I production by pDCs and monocytes is a promising strategy to dampen inflammation in severe influenza cases without losing antiviral protection by epithelial-derived IFN-I.

Among various strategies to block TLR signaling, oligodeoxyribonucleotides (ODNs) antagonizing TLR activation are the most clinically advanced, with several candidates having successfully passed phase 2 trials (Balak et al., 2017; Fulmer, 2010; Kimball et al., 2013; Robbins et al., 2007; Suárez-Fariñas, M., J. Belasco, J. Fuentes-Duculan, T. Sullivan, R. Arbeit, and J. Krueger. 2013. American College of Rheumatology (ACR) 13. Abstr. 1171). The ODN immunoregulatory sequence 661 (IRS661) contains the sequence 5′-TGC​TTG​CAA​GCT​TGC​AAG​CA-3′ and specifically targets TLR7 in vitro and in vivo (Barrat et al., 2005; Pawar et al., 2007). TLR7 inhibition is thought to be both sequence dependent (through the 5′ TGC motif; Barrat et al., 2005) and sequence independent (through its phosphorothioate backbone; Jurk et al., 2006; Lenert et al., 2009). We hypothesized that IRS661 would display beneficial anti-inflammatory effects in a mouse model of severe influenza.

## Results and discussion

### IRS661 inhibits TLR7-mediated production of cytokines in vitro

**Figure 1.**
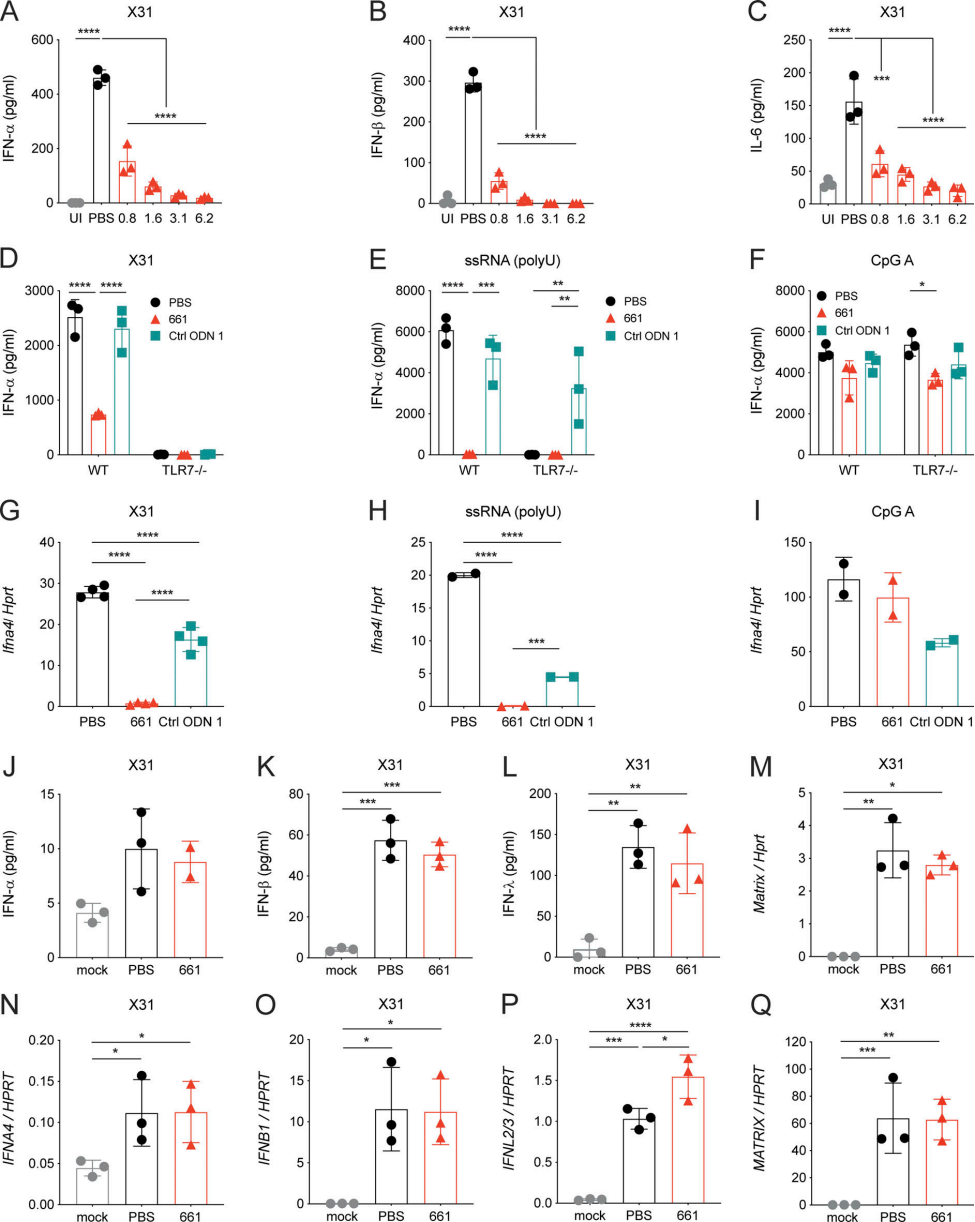
**IRS661 inhibits TLR7-mediated production of cytokines in immune cells.** **(A–C)** Bone marrow–derived pDCs from WT 129S6 mice were stimulated with influenza virus X31 (MOI 0.35) in the presence of PBS or increasing amounts of IRS661 (0.8–6.2 µM) or left uninfected (UI). IFN-α (A), IFN-β (B), and IL-6 (C) levels were measured in the supernatant by ELISA 24 h after stimulus. **(D–I)** Bone marrow–derived pDCs from WT or TLR7 KO C57BL/6 mice were stimulated with X31 (MOI 1; D and G); ssRNA polyU (1 µg/ml) complexed with LyoVec (E and H); or CpG-A 2216 (6 µg/ml; F and I) in the presence of PBS, IRS661 (1 µM), or control ODN 1 from TIB Technologies (1 µM). IFN-α levels were measured in the supernatant by ELISA 24 h after stimulation (D–F), and cellular messenger RNA levels were measured by RT-qPCR 6 h after stimulation (G–I). **(J–Q)** Airway epithelial cell cultures from C57BL/6 mice (J–M) or healthy human donors (N–Q) were infected with X31 (MOI 0.5) in the presence of PBS or IRS661 (1 µM) or left uninfected (mock). IFN-α (J and N), IFN-β (K and O), IFN-λ (L and P), and influenza *matrix* levels (M and Q) were measured in the supernatant by ELISA (J–L) or RT-qPCR in cell lysates (M–Q) 24 h later. Data are representative of ≥2 independent experiments where *n* = 3–12 mice or 3 human donors, and each symbol represents a biological replicate. Statistical analysis was performed using ordinary one-way ANOVA with Tukey’s multiple comparisons test (A–C and G–Q) or two-way ANOVA with Tukey’s multiple comparisons test (D–F). *, P < 0.05; **, P < 0.01; ***, P < 0.001; ****, P < 0.0001.

To explore the potential of IRS661 to block influenza-induced IFN-I production by pDCs, we first stimulated bone marrow–derived pDCs with influenza virus (X31) in the presence of PBS or increasing doses of IRS661. IRS661 concentrations as low as 0.8 µM induced a fourfold reduction, while 1.6 µM led to a sevenfold reduction of IFN-α amounts (Fig. 1 A). This latter concentration completely inhibits IFN-β production (Fig. 1 B) and significantly reduces IL-6 levels (Fig. 1 C). Accordingly, we chose to use 1 µM in the subsequent in vitro experiments. Comparing bone marrow–derived pDCs from WT and TLR7 KO mice stimulated with influenza virus (Fig. 1 D), we confirmed the strict dependence of pDCs on TLR7 for IFN-I production, consistent with previously published results (Diebold et al., 2004; Lund et al., 2004). IFN-α production was abrogated by IRS661 when the cells were stimulated with a prototypic TLR7 agonist (single-stranded RNA polyuridine [ssRNA polyU]; Fig. 1 E), but not with a TLR9 agonist (CpG-A; Fig. 1 F). IFN-α gene induction was also significantly decreased by IRS661 following X31 infection (Fig. 1 G) and ssRNA treatment (Fig. 1 H), but not after CpG-A treatment (Fig. 1 I), confirming the strict specificity of IRS661 for TLR7 without effects on TLR9 (Barrat et al., 2005). Human and murine epithelial cells employ RIG-I but not TLR7 to sense influenza virus infection and produce IFNs in response; therefore, IRS661 does not inhibit the production of IFN-I and IFN-III by murine (Fig. 1, J–M) or human (Fig. 1, N–Q) epithelia. Virus replication was unchanged in these cultures, excluding direct antiviral action of IRS661 (Fig. 1, M and Q). In conclusion, IRS661 inhibits TLR7-dependent production of IFN-I by immune cells but preserves the production of epithelial-derived IFNs.

### Control ODN 1 does not affect TLR7-mediated IFN-I induction but shows other activities

**Figure S1.**
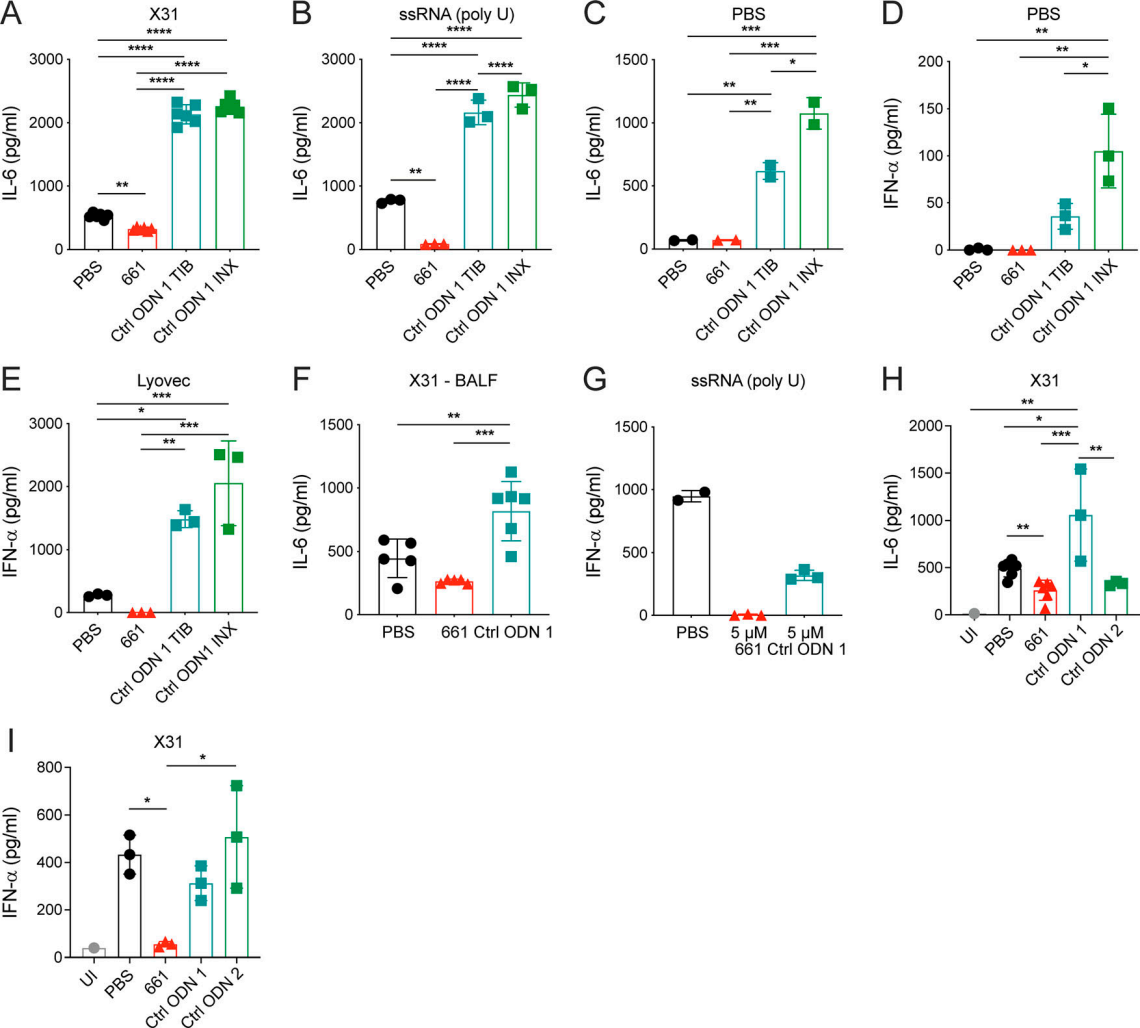
**The control ODN 1 is not inert but induces cytokines both in vitro and in vivo.** **(A–E and G–I)** Bone marrow–derived pDCs from WT C57BL/6 mice (A–E and G) or WT 129S7 mice (H and I) were stimulated with influenza virus X31 (MOI 0.5; A, H, and I); ssRNA polyU (1 µg/ml) complexed with LyoVec (B and G); LyoVec alone (E) or left unstimulated (PBS in C and D; UI in H and I) in the presence of PBS, IRS661, control ODN 1 from TIB Technologies (Ctrl ODN 1/Ctrl ODN 1 TIB) or Innaxon (Ctrl ODN 1 INX) or control ODN 2, all at 1 µM (except G: 5 µM). IL-6 (A–C, F, and H) and IFN-α (D, E, G, and I) levels were measured in the supernatant by ELISA 24 h after stimulation. Data are pooled from at least two independent experiments. **(F)** 129S7 mice were infected intranasally with 1,000 TCID_50_ X31. 48 h after infection, PBS, IRS661 (15 nmol/mouse), or control ODN 1 (15 nmol/mouse) was administered intranasally at 30 µl. 8 h later, mice were euthanized, and IL-6 levels were measured in the BALF by ELISA. Data are representative of ≥2 independent experiments with *n* = 5–6 mice per group. Each symbol represents a biological replicate. Statistical analysis was performed using ordinary one-way ANOVA with Tukey’s multiple comparisons test. *, P < 0.05; **, P < 0.01; ***, P < 0.001; ****, P < 0.0001.

The oligodeoxynucleotide 5′-TCC​TGC​AGG​TTA​AGT-3′ (Ctrl ODN 1), used in most studies as a control for IRS661 (Barrat et al., 2005; Fabié et al., 2018; Lannes et al., 2012; Python et al., 2013; Sun et al., 2009; Takahashi et al., 2010; Yin et al., 2014), was described as “inactive.” We tested the Ctrl ODN 1 next to IRS661 and indeed found little effect on IFN-I production by influenza virus or TRL7 and TLR9 agonists at the concentration of 1 µM (Fig. 1, D–F). Unexpectedly, when murine pDCs were stimulated with influenza virus or ssRNA polyU, addition of Ctrl ODN 1 (1 µM) increased IL-6 production (Fig. S1, A and B). Effects were found with Ctrl ODN 1 made by two different companies: TIB MOLBIOL (Ctrl ODN 1 TIB) and Innaxon (Ctrl ODN 1 INX, certified endotoxin-free; Fig. S1, A–E). To our great surprise, Ctrl ODN 1 on its own, when added to unstimulated cells, triggered strong IL-6 (Fig. S1 C) and moderate IFN-α (Fig. S1 D) production. In addition, Ctrl ODN 1 induced IFN-α production in pDCs when treated with LyoVec (Figs. 1 E and S1 E). The pro–IL-6 effect of Ctrl ODN 1 was confirmed in vivo. Indeed, influenza virus–infected mice treated with Ctrl ODN 1 exhibited higher levels of the cytokine in their bronchoalveolar lavage fluid (BALF) 8 h after Ctrl ODN 1 administration (Fig. S1 F). Moreover, Ctrl ODN 1 is a 15-mer (vs. 20 for IRS661) with a phosphorothioate backbone, therefore potentially interfering with TLR7 signaling as previously suggested (Jurk et al., 2006; Lenert et al., 2009). This was the case when Ctrl ODN 1 was used at high concentrations (5 µM; Fig. S1 G). For these reasons, we decided not to use ODN 1 as control treatment in in vivo experiments. We designed a new control ODN 2 (5′-GCT​CCT​AGT​AGA​GAT​CTC​AG-3′), which is a scrambled version of IRS661. Treatment with this Ctrl ODN 2 did not alter IL-6 or IFN-α levels in X31-stimulated 129S7 pDC cultures in comparison with PBS (Fig. S1, H and I).

### Local administration of IRS661 reduces levels of proinflammatory cells and cytokines in a mouse model of severe influenza

**Figure S2.**
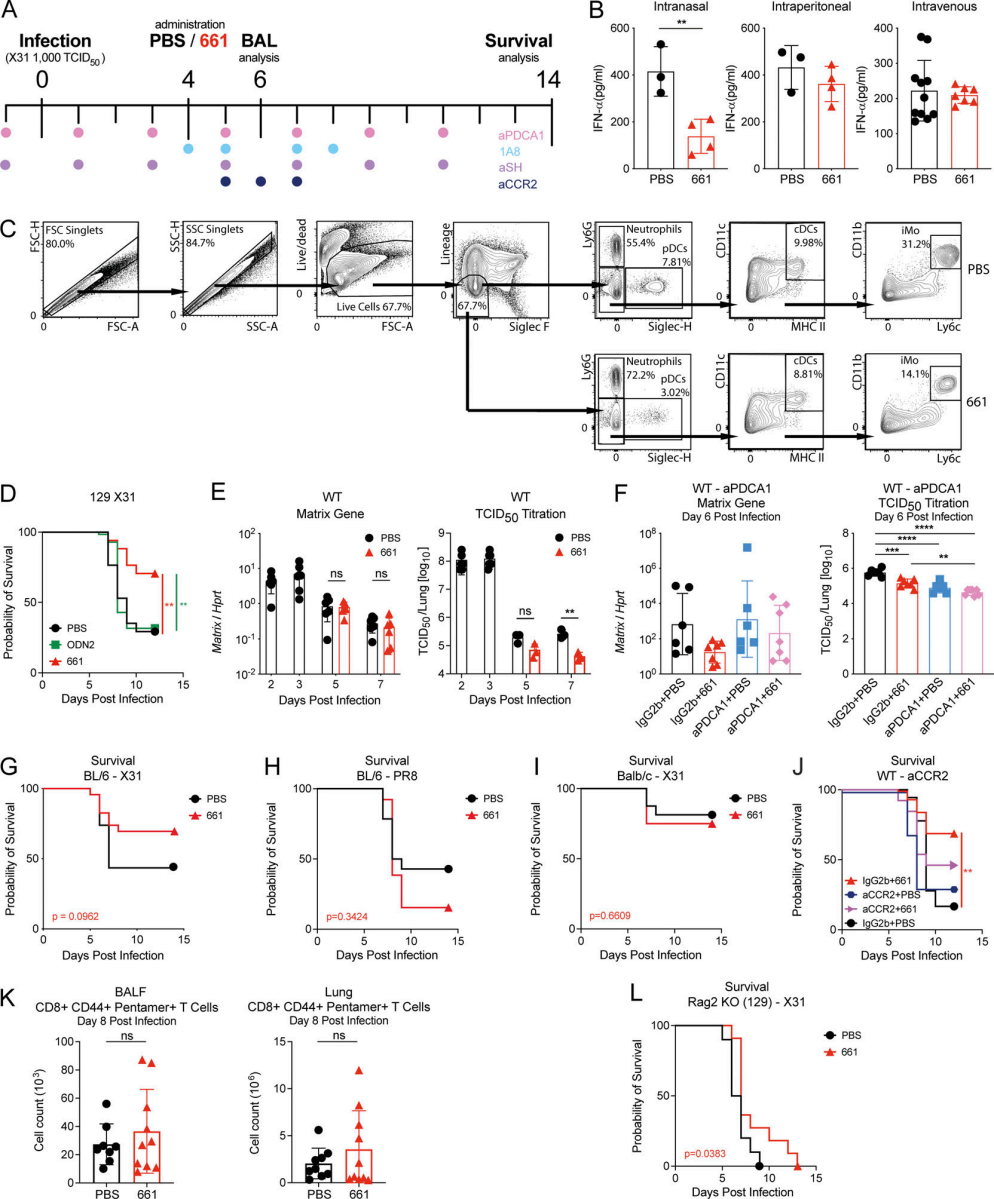
**Mechanism of action of IRS661.** **(A)** Schematic representation of the experimental setup. Mice were infected intranasally with 1,000 TCID_50_ of influenza virus X31 and treated with IRS661 or PBS intranasally (15 nmol in 30 µl) on day 4 after infection. Usually, BALF was isolated on day 6 after infection. Colored dots mark days when mice were treated with αPDCA-1 (200–500 µg), 1A8 (150–300 µg), αSiglec-H (200–500 µg), or αCCR2 (20 µg) depletion antibody i.p. **(B)** WT 129S7 mice were infected with X31 and treated with IRS661 or PBS intranasally (15 nmol in 30 µl), i.p. (50 nmol in 100 µl), or intravenously (50 nmol in 100 µl) 4 d later. IFN-α concentrations were quantified by ELISA in BALF taken on day 5 after infection. *n* = 3–11 mice per group. **(C)** Flow cytometry plots representing the gating strategy for cell analysis in Fig. 2 B. Lineage channel includes CD3, CD19, and NKp46. FSC, forward scatter; SSC, side scatter. **(D)** WT 129S7 mice were infected with 1,000 TCID_50_ X31 and treated with IRS661, control ODN 2, or PBS intranasally. Survival was monitored for 14 d. *n* = 17–18 mice per group. **(E and F)** Viral loads in lungs of X31-infected mice treated with IRS661 or PBS (E; *n* = 3–7 mice per group) and/or aPDCA-1 antibody (F; *n* = 6–7 mice per group) were determined by RT-qPCR or TCID_50_ assay at days 2, 3, 5, and 7 (E) or day 6 (F) after infection. **(G)** WT C57BL/6 mice were infected with 10,000 TCID_50_ X31 and treated with IRS661 or PBS intranasally. Survival was monitored for 14 d. *n* = 23 mice per group. **(H)** WT C57BL/6 mice were infected with 5 TCID_50_ PR8 and treated with IRS661 or PBS intranasally. Survival was monitored for 14 d. *n* = 13–14 mice per group. **(I)** WT Balb/c mice were infected with 10,000 TCID_50_ X31 and treated with IRS661 or PBS intranasally. Survival was monitored for 14 d. *n* = 16 mice per group. **(J)** WT 129S7 mice were infected and treated with IRS661 or PBS and αCCR2 antibody or isotype control as depicted in A. Survival was monitored for 14 d. *n* = 18–20 mice per group. **(K)** WT 129S7 mice were infected with X31 and treated with IRS661 or PBS. BALF and lung were isolated on day 8 after infection and checked for CD44^+^ Pentamer^+^ CD8^+^ T cells by flow cytometry. *n* = 8–10 mice per group. **(L)** Rag-2 KO (129) mice were infected with 750 TCID_50_ X31 and treated with IRS661 or PBS intranasally. Survival was monitored for 14 d. *n* = 10–11 mice per group. Data are pooled from at least two independent experiments, and each symbol in the bar charts represents a biological replicate. Statistical analysis was performed using unpaired *t* test (B and K), log-rank test (D, G–J, and L), 2-way ANOVA with Tukey’s multiple comparisons test (E), or ordinary one-way ANOVA with Tukey’s multiple comparisons test (F). **, P < 0.01; ***, P < 0.001; ****, P < 0.0001.

**Figure 2.**
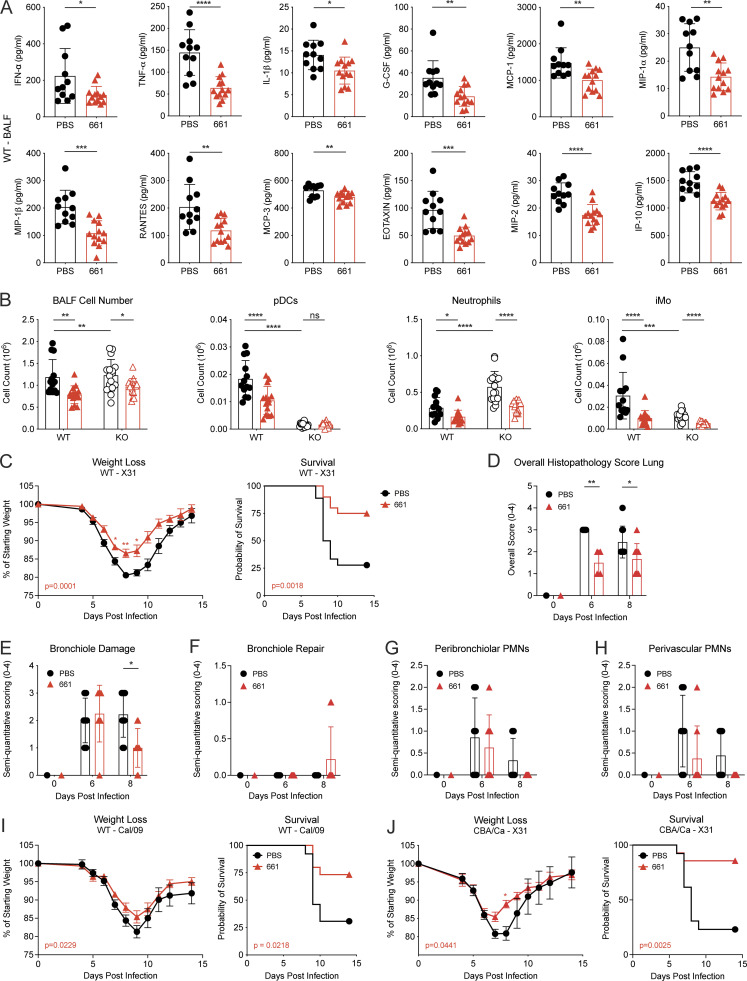
**IRS661 decreases IFN-I and proinflammatory cytokine production in vivo during influenza infection and reduces cell recruitment, tissue damage, and morbidity.** **(A–H)** WT 129S7 mice and IFNAR1 KO 129S7 mice (B) were infected intranasally with 1,000 median tissue culture infectious dose (TCID_50_) influenza virus strain X31 and treated intranasally with IRS661 (15 nmol) or PBS in 30 µl 4 d later. **(A)** IFN-α and specified proinflammatory cytokine concentrations were quantified in BALF by multiplex on day 6 after infection. *n* = 11–13 mice per group. **(B)** Total cells, pDCs, neutrophils, and inflammatory monocyte (iMo) populations were quantified by flow cytometry in BALF taken from WT and IFNAR KO 129S7 mice on day 6 after infection. *n* = 13–16 mice per group. Black symbols, PBS; red symbols, IRS661. **(C)** Weight loss and survival of WT mice were monitored for 14 d. *n* = 18–20 mice per group. **(D–H)** Histopathological analysis of H&E-stained lung sections on days 6 and 8 after infection. *n* = 4–9 mice per group. **(I)** Weight loss and survival of WT 129S7 mice infected with 100 TCID_50_ influenza virus strain Cal/09 and treated intranasally with IRS661 (15 nmol) or PBS in 30 µl 4 d later. *n* = 13–15 mice per group. **(J)** Weight loss and survival of WT CBA/Ca mice infected with 1,000 TCID_50_ X31 and treated intranasally with IRS661 (15 nmol) or PBS in 30 µl 4 d later. *n* = 13–14 mice per group. All data are pooled from at least two independent experiments. Each symbol in the bar charts represents a biological replicate. Statistical analysis was performed using unpaired *t* test (A), two-way ANOVA with Tukey’s multiple comparisons test (B), mixed-effects analysis with Sidak’s multiple comparisons test (D–H and weight loss in C, I, and J) or log-rank test (survival in C, I, and J). *, P < 0.05; **, P < 0.01; ***, P < 0.001; ****, P < 0.0001.

To understand if IRS661 can alleviate influenza-induced CS and the morbidity and mortality it drives, we used the highly influenza-susceptible 129Sv mouse strain, whose mortality was shown to be IFN-I dependent (Boivin et al., 2012; Boon et al., 2011; Davidson et al., 2014; Srivastava et al., 2009; Zhou et al., 2016). Mice were infected with influenza virus and treated with IRS661 or PBS 4 d later (Fig. S2 A). 6 d after infection, mice were euthanized, and cytokines were measured in the BALF (Fig. 2 A). IRS661 was able to significantly reduce the levels of IFN-I and a wide range of cytokines (TNF-α, IL-1β, and G-CSF) and chemokines. When comparing different administration routes, only intranasal treatment led to a significant decrease of IFN-α levels in the BALF (Fig. S2 B). Intranasal administration was the preferred route used by many other groups to deliver phosphorothioated oligodeoxynucleotides (Campbell et al., 2014; Kim et al., 2019; Pesce et al., 2010) or oligonucleotides (Finotto et al., 2001; Tomaru et al., 2017; Torres et al., 2008) to the respiratory tract. When looking at inflammatory cell types in the BALF, IRS661 significantly decreased the total number of cells, pDCs, inflammatory monocytes, and neutrophils (Fig. 2 B; gating strategy in Fig. S2 C). Treatment with IRS661 as late as 4 d after influenza infection led to a decrease in weight loss and mortality as compared with PBS or control ODN 2 treatment (Figs. 2 C and S2 D), with minor changes of viral load in the lung (Fig. S2, E and F). Overall lung damage was significantly reduced by IRS661 (Fig. 2 D), with less bronchial erosion and ulceration detectable as well as trends toward increased regeneration (Fig. 2, E and F). Reduced neutrophil infiltration was confirmed by the trends found in histopathological analysis (Fig. 2, G and H). Of note, IRS661 treatment also improved disease in 129S7 mice infected with the pandemic influenza strain Cal/09 (Fig. 2 I), and in CBA/Ca mice, which exhibit strong IFN responses and high susceptibility to X31 infection (Fig. 2 J). In contrast, in low IFN responder strains such as C57BL/6 and Balb/c mice, IRS661 treatment did not improve resistance to influenza infection (Fig. S2, G–I) which is in line with Fang et al. (2011), who showed that a microsatellite ODN capable of inhibiting TLR7/9 failed to reduce acute lung inflammatory injury in Balb/c mice. Thus, the IRS661-mediated blockade of cytokine production reduces morbidity and mortality without compromising viral control in mouse strains highly prone to IFN responses and susceptible to influenza.

### Protective effects of IRS661 are mediated by pDCs

**Figure 3.**
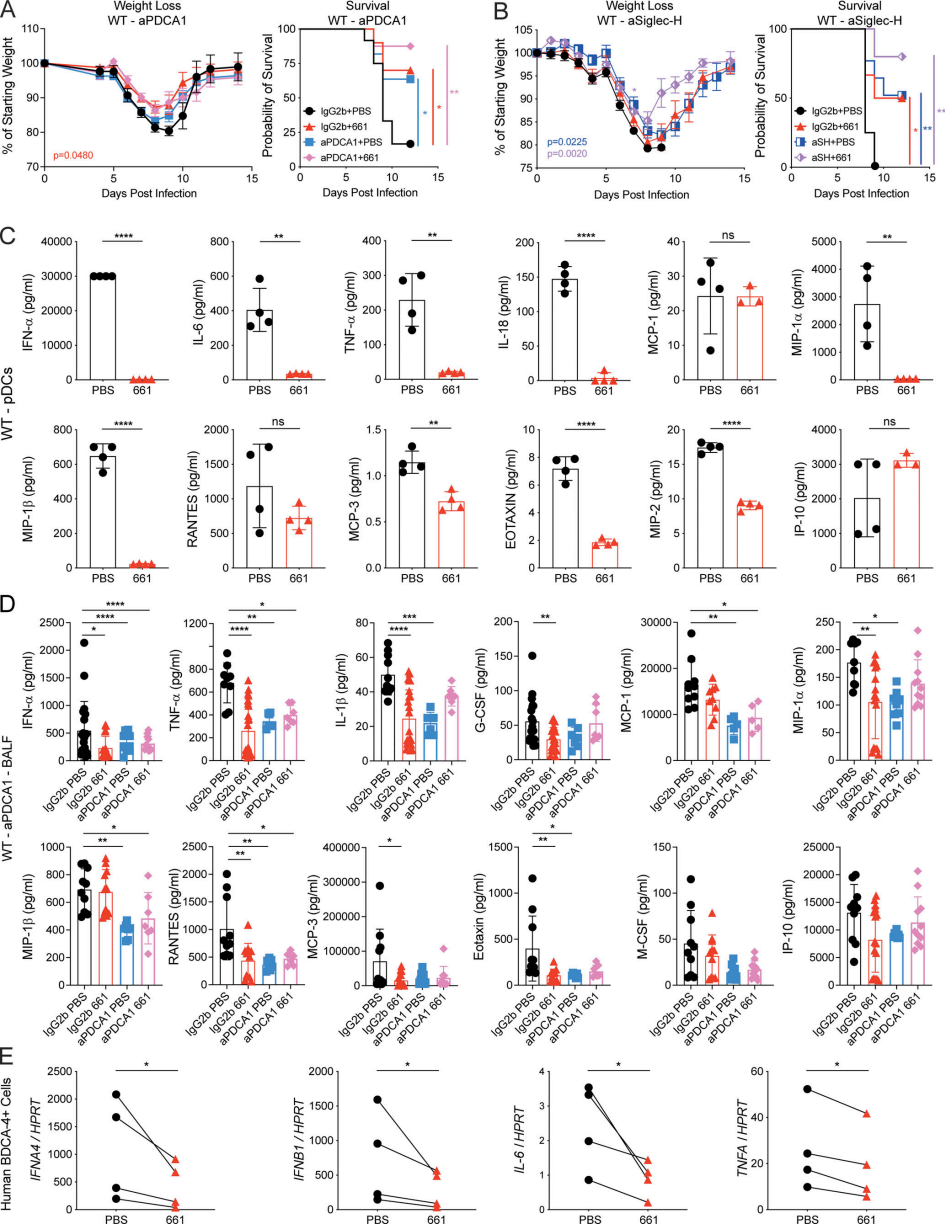
**IRS661 effects are mainly mediated by pDCs in WT mice.** **(A and B)** WT 129S7 mice were infected with 1,000 TCID_50_ of X31 and treated intranasally with IRS661 (15 nmol) or PBS in 30 µl 4 d later. Mice were treated with depleting mAb αPDCA-1 (A) or αSiglec-H (B) or isotype control as depicted in Fig. S2 A. Weight loss and mortality were measured for 14 d. Data are pooled from two independent experiments with *n* = 8–12 mice per group (A) and *n* = 6–8 mice per group (B). **(C)** Bone marrow–derived pDCs from WT 129S7 mice (*n* = 4) were stimulated with X31 (MOI 3) in the presence of PBS or IRS661 (1 µM). IFN-α and specified proinflammatory cytokine concentrations were quantified in supernatants by multiplex 24 h after stimulation. **(D)** WT 129S7 mice were infected and treated with IRS661 or PBS and αPDCA-1 antibody or isotype control as depicted in Fig. S2 A. Specified cytokines were quantified in BALF by multiplex on day 6 after infection. *n* = 7–21 mice per group. **(E)** Human peripheral blood BDCA-4^+^ cells from four different donors were stimulated with X31 (MOI 1) in the presence of PBS or IRS661 (1 µM). IFN-α, IFN-β, IL-6, and TNFα mRNA levels were quantified from cell lysate by RT-qPCR 24 h after stimulation. Data are pooled from two independent experiments. Each symbol in C and E represents a biological replicate. Statistical analysis was performed using mixed-effects analysis with Sidak’s multiple comparisons test (weight loss in A and B), log-rank test (survival in A and B), unpaired *t* test (C), ordinary one-way ANOVA with Tukey’s multiple comparisons test (D), or paired *t* test (E). *, P < 0.05; **, P < 0.01; ***, P < 0.001; ****, P < 0.0001.

Given the pivotal proinflammatory role of PDCA-1–positive cells, including pDCs and activated monocytes, in influenza-induced immunopathology (Davidson et al., 2014), we tested whether these cells are the main target of IRS661 in WT mice. Influenza virus–infected mice were treated with IRS661 or PBS, while PDCA-1–expressing cells were depleted (Fig. 3 A). In line with previous observations, cell depletion using the αPDCA-1 mAb significantly improved survival. This was accompanied by an αPDCA-1–dependent reduction in viral loads, observed in mice treated or not with IRS661 (Fig. S2 F). In the absence of pDCs and PDCA-1–expressing cells, IRS661 does not further increase survival (Fig. 3 A) or decrease virus titers (Fig. S2 F). We confirmed these findings by treating mice with αSiglec-H mAb (Fig. 3 B), which depletes pDCs more specifically than αPDCA-1 treatment (Blasius et al., 2006). Furthermore, depletion of monocytes in WT mice did not improve survival significantly (Fig. S2 J). These results suggest that pDCs are indeed among the main targets of IRS661 in WT mice. To characterize globally the action of IRS661 on murine pDCs, we measured how IRS661 treatment modifies the levels of multiple cytokines produced by bone marrow–derived pDCs stimulated by influenza virus in vitro (Fig. 3 C). Apart from MCP-1, RANTES, and IP-10, the proteins measured in the cell culture supernatant were decreased by IRS661 treatment similarly to what was observed in the BALF of the flu-infected, IRS661-treated mice (Fig. 2 A). Of note, cytokine levels in the BALF of IRS66-treated mice were reduced to the same extent as in αPDCA-1–treated animals (Fig. 3 D). Therefore, IRS661 blocks TLR7-dependent, pDC-driven inflammation that causes severe influenza. pDCs differ substantially across species (Auray et al., 2016). Therefore, we tested whether IRS661 had the same anti-inflammatory effect on human cells. pDCs purified from blood of four different donors were stimulated with live X31 in the presence or absence of IRS661 (Fig. 3 E). IRS661 diminished the influenza virus–induced transcripts of IFN-I and the cytokines IL-6 and TNF-α to an extent similar to that found in murine pDCs and in accordance with previous reports (Barrat et al., 2005; Wang et al., 2006).

To assess involvement of adaptive responses in IRS661-mediated protection, we evaluated numbers of influenza-specific CD8^+^ T cells by flow cytometry on day 8 after infection and found no effect of IRS661 treatment (Fig. S2 K). Furthermore, in Rag2 KO (129) mice, which are devoid of adaptive immunity, IRS661 treatment increased median survival (Fig. S2 L), confirming that IRS661-mediated protection is independent of adaptive immunity.

### IFN-independent morbidity is reduced by IRS661

**Figure 4.**
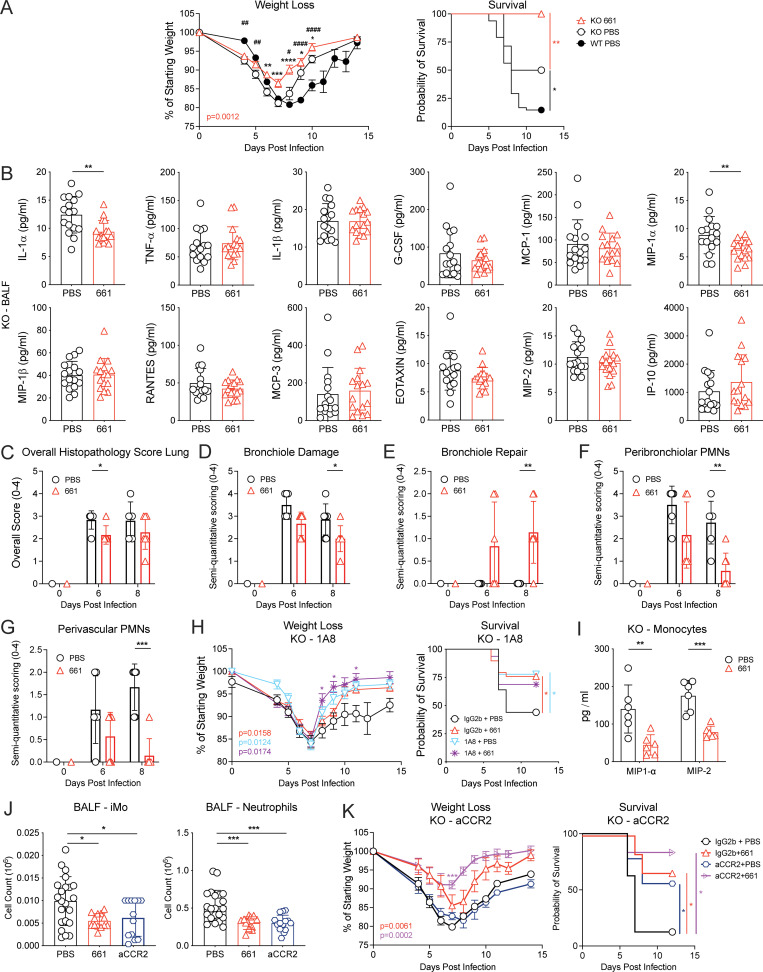
**IFN-I–independent effects of IRS661 are mediated via a monocyte–neutrophil axis.** **(A–H, J, and K)** IFNAR1 KO 129S7 mice or WT 129S7 mice (A) were infected with 1,000 TCID_50_ X31 and treated with IRS661 or PBS intranasally on day 4. Additionally, mice were treated with 1A8 (H), αCCR2 depletion antibody (J and K), or isotype control as shown in Fig. S2 A. **(A)** Weight loss and survival were monitored for 14 d. *n* = 13–14 mice for KO groups, 48 mice for WT group. For weight loss symbols, refer to single time points with KO PBS vs. WT PBS: ^#^, P < 0.05; ^##^, P < 0.01; ^####^, P < 0.0001; KO PBS vs. KO IRS661: *, P < 0.05; **, P < 0.01; ***, P < 0.001; ****, P < 0.0001. **(B)** Specified cytokines were quantified in BALF by multiplex on day 6 after infection. *n* = 16 mice per group. **(C–G)** Histopathologic analysis of H&E-stained lung sections on days 6 and 8 after infection. *n* = 5–7 mice per group. PMN, polymorphonuclear leukocyte. **(H)** Weight loss and mortality were measured for 14 d. *n* = 18–29 mice per group. **(I)** Bone marrow–derived monocytes from IFNAR1 KO 129S7 mice were stimulated with influenza virus X31 (MOI 1) in the presence of PBS or IRS661 (1 µM). MIP-1α and MIP2 concentrations were quantified in supernatant by multiplex 24 h after stimulation. *n* = 6 mice per group. **(J)** Inflammatory monocytes (iMo) and neutrophils were quantified by flow cytometry in BALF taken on day 6 after infection. *n* = 13–22 mice per group. **(K)** Weight loss and survival were monitored for 14 d. *n* = 6–9 mice per group. All data are pooled from at least two independent experiments, and each symbol in bar charts represents a biological replicate. Statistical analysis was performed using mixed-effects analysis with Sidak’s multiple comparisons test (C–G and weight loss in A, H, and K), log-rank test (survival in A, H, and K), unpaired *t* test (B), or two-way ANOVA with Sidak’s multiple comparisons test (I) or Tukey’s multiple comparisons test (J). *, P < 0.05; **, P < 0.01; ***, P < 0.001; ****, P < 0.0001.

**Figure S3.**
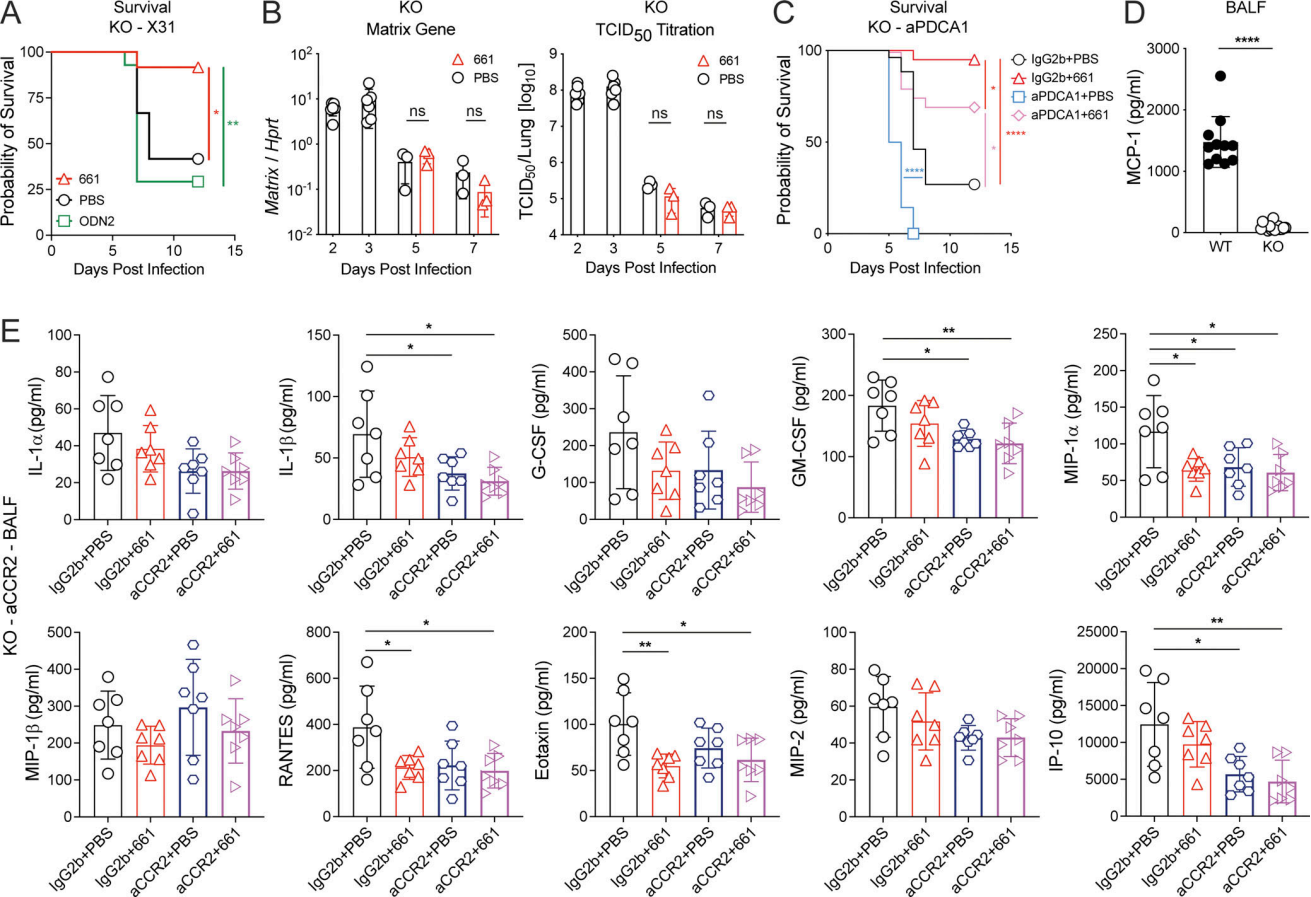
**IFN-I–independent disease improvement by IRS661 is mediated via a monocyte–neutrophil axis.** **(A–E)** IFNAR1 KO 129S7 mice and WT 129S7 mice (D) were infected with 1,000 TCID_50_ X31 and treated with IRS661, control ODN 2, or PBS intranasally on day 4. Additionally, mice were treated with αPDCA-1–depleting (C) or αCCR2-depleting (E) antibodies or isotype control as shown in Fig. S2 A. **(A)** Survival was monitored for 14 d. *n* = 11–12 mice per group. **(B)** Viral load was measured by RT-qPCR or TCID_50_ assay on days 2, 3, 5, and 7 after infection. *n* = 3–6 mice per group. **(C)** Survival was monitored for 14 d. *n* = 20–26 mice per group. **(D)** MCP-1 concentration in BALF was quantified by multiplex on day 6 after infection. *n* = 11–16 mice per group. **(E)** Specified cytokines in BALF were quantified by multiplex on day 6 after infection. *n* = 7 mice per group. All data are pooled from at least two independent experiments, and each symbol in the bar charts represents a biological replicate. Statistical analysis was performed using log-rank test (A and C), two-way ANOVA with Tukey’s multiple comparisons test (B), unpaired *t* test (D), or ordinary one-way ANOVA with Tukey’s multiple comparisons test (E). *, P < 0.05; **, P < 0.01; ****, P < 0.0001.

IFN-I is not always the driver of CS in severe respiratory infection, as exemplified by COVID-19 (Hadjadj et al., 2020). In addition, IFN responses to influenza infection are reduced in the elderly (Pillai et al., 2016), yet the elderly frequently suffer from severe influenza associated with immunopathology. We therefore tested whether there are IFN-I–independent effects of TLR7 antagonism during severe influenza virus infection. First, we confirmed that removal of IFN-I signaling by deleting the IFNAR chain *Ifnar1* improved weight loss and survival in our influenza model (Fig. 4 A), as described previously (Davidson et al., 2014). Notably, IRS661 treatment further reduced morbidity and mortality of influenza infection in IFNAR1-deficient mice (Figs. 4 A and S3 A). IRS661 treatment had no effect on viral load (Fig. S3 B) but reduced levels of the cytokines IL-1α and Mip-1α in the BALF of IFNAR1 KO mice (Fig. 4 B), in association with less inflammatory monocyte and neutrophil infiltration, while pDC numbers were very low in IFNAR1 KO mice and did not change upon treatment (Fig. 2 B). We confirmed that pDCs are not the driving force of morbidity and the target of IRS661 by depleting these cells from IFNAR1 KO mice without recapitulating the improvement seen with IRS661 treatment (Fig. S3 C). Lungs of IRS661-treated IFNAR1 KO mice showed a lower histopathology score and improved repair (Fig. 4, C–E), in line with overall reduced morbidity.

Our results indicate that IRS661 is also beneficial against severe influenza characterized by absence of pDCs and IFN-I signaling. We therefore asked which cells may be responsible for the immunopathology in IFNAR1 KO mice and may be targeted by IRS661. IFNAR1 KO mice have lower monocyte numbers in BALF than WT mice (Fig. 2 B), likely due to the near-absence of MCP-1 in IFNAR1-deficient mice (Fig. S3 D). In contrast, more neutrophils are recruited into the lung in the absence of IFN-I signaling (Fig. 2 B; Davidson et al., 2014), and IRS661 treatment reduces neutrophil numbers significantly (Fig. 2 B and Fig. 4, F and G). Neutrophils have been reported to have both beneficial (Tak et al., 2018; Tate et al., 2009; Tate et al., 2011) and detrimental (Brandes et al., 2013; Narasaraju et al., 2011; Trompette et al., 2018; Vidy et al., 2016; Zhu et al., 2019) potential during influenza. Therefore, we tested if the reduction of neutrophil numbers explains the protection conferred by IRS661 treatment. Neutrophils were depleted by repeated injections of an αLy6G monoclonal antibody (1A8; Fig. S2 A). Neutrophil depletion greatly improved survival of IFNAR1 KO mice, and in the absence of neutrophils, IRS661 treatment had no further beneficial effect (Fig. 4 H). We next tested whether neutrophils are the direct target of IRS661 treatment, but were not able to elicit a response to X31 stimulation from mouse neutrophils (not depicted), consistent with the 10-fold lower level of TLR7 expression on murine neutrophils as compared with monocytes (ImmGen database; Heng et al., 2008). Monocytes contribute to neutrophil recruitment during influenza virus infection (Trompette et al., 2018) via the production of chemoattractants such as Mip-1α, Mip-2, and CXCL-1 (McColl and Clark-Lewis, 1999; Reichel et al., 2012; Seo et al., 2011; Shahangian et al., 2009). We found that monocytes purified from IFNAR1 KO bone marrow produced high amounts of the neutrophil chemoattractants Mip-1α and Mip-2 in response to X31 that were reduced by IRS661 treatment (Fig. 4 I), in line with IRS661-mediated Mip-1α reduction in the BALF of IFNAR1 KO mice (Fig. 4 B). Moreover, αCCR2 antibody–mediated monocyte depletion in IFNAR1 KO mice also reduced Mip-1α (Fig. S3 E) and neutrophil recruitment into BALF (Fig. 4 J), resulting in increased survival, comparable to the IRS661-treated control group (Fig. 4 K). Our findings are in line with a previous report of increased numbers of monocytes and neutrophils upon sustained TLR7 stimulation, independently of IFN-I (Baenziger et al., 2009). We conclude that in influenza responses not dominated by IFN-I, high neutrophil numbers contribute to immunopathology, and we find that IRS661 treatment reduces the monocyte-dependent infiltration of neutrophils into the lung.

### Concluding remarks

Our results demonstrate that a single intranasal administration of the oligodeoxynucleotide IRS661, as late as 4 d after influenza infection, reduces damage driven by proinflammatory immune cells while conserving the protective IFN response of infected lung epithelia. IRS661 is a host-directed treatment that is protective in diverse settings of anti-influenza responses: in high IFN-I responders, IRS661 blocks pDC-dependent IFN-I production that leads to monocyte-driven immunopathology (Davidson et al., 2014; Högner et al., 2013). In the absence of IFN-I signaling, IRS661 reduces the virus-induced, TLR7-dependent production of neutrophil chemoattractants by monocytes, leading to lower lung neutrophil numbers and thereby reduced immunopathology and mortality. Therefore, TLR7 blockade has therapeutic potential in diverse contexts of pulmonary inflammation and CS during influenza virus infection.

Host-directed interventions to reduce disease severity in infection need to take into account the heterogeneity of the host immune responses. Examples of this heterogeneity are the different characteristics of the CS in several viral and bacterial infections. The CS found in non-human primates infected with highly pathogenic H5N1 influenza virus was dominated by IFN-I (Baskin et al., 2009), while influenza causes immunopathology in the elderly despite a subdued IFN-I response (Pillai et al., 2016). Similarly, the CS in bacterial sepsis was treated successfully with the IL-1 receptor antagonist anakinra in patients with features of the macrophage activation syndrome, but not in patients without these features, whose CS was likely driven by cytokines other than IL-1 (Shakoory et al., 2016). We show here that IRS661 treatment improves influenza severity in two rather diverse settings, suggesting that differing host immune responses can be accommodated by this treatment.

The protective effect of IRS661 lies in the inhibition of TLR7 signaling in our model of immunopathology in influenza-susceptible 129 mice. The role of TLR8 is controversial in mice, but in humans, TLR8 is also able to sense viral RNA (de Marcken et al., 2019; Guiducci et al., 2013; Heil et al., 2004). Therefore, dual inhibition of TLR7/TLR8 may be required in humans to obtain a beneficial effect as demonstrated here. However, we show here that IRS661 treatment of human pDCs efficiently blocked IFN production induced by influenza virus. While the TLR7 KO allele was never bred into the 129 background, it was shown in C57BL/6 mice that TLR7 deficiency does not impact influenza virus control but reduces proinflammatory cytokines (Jeisy-Scott et al., 2011). TLR7 KO mice also recover weight more slowly after infection, a phenomenon that we do not observe. This discrepancy may depend on permanent loss of TLR7 as compared with temporary blockade after infection onset in our experiments. However, influenza virus infection in C57BL/6 does not lead to the massive CS observed in 129 mice (Davidson et al., 2014) and therefore could not be used as a model of severe flu in our experiments. Prophylactic or early use of TLR7 agonists is efficient against flu (To et al., 2019; Wu et al., 2007) and respiratory syncytial virus (Salinas et al., 2020), as induction of IFN-I before infection efficiently blunts viral replication at its onset (Davidson et al., 2016). While such prophylaxis for uninfected people is clinically unrealistic, we show that administration of the TLR7 antagonist IRS661 4 d after infection cuts down the otherwise prolonged production of IFN-I, inflammatory cytokines, and chemokines. This scenario better corresponds to the reality of most influenza patients usually seeking care >2 d after the onset of symptoms (Puig-Barberà et al., 2016). However, as TLR7-mediated bronchodilation was described to be blocked by IRS661 (Drake et al., 2013), care must be taken when TLR7 antagonists are considered for therapy in respiratory infections.

While inhibition of other TLRs (Piao et al., 2015; Shirey et al., 2013) was shown to efficiently reduce lethality during influenza infection in mice, TLR7 inhibition has mainly been investigated in the context of autoimmune diseases (Barrat and Coffman, 2008). We demonstrate here for the first time that specific TLR7 inhibition well into the course of infection efficiently dampens the CS associated with influenza. Infection with SARS-CoV-2 has been shown to cause cytokine release syndrome in patients with severe Covid-19 (Moore and June, 2020). It is therefore crucial to test if IRS661 could mitigate coronavirus-induced inflammation as effectively as demonstrated here for influenza-related CS.

## Materials and methods

### Mice

129S6, 129S7, IFNAR1 KO, and Rag2 KO mice on 129S7 background; C57BL/6J and TLR7 KO mice on C57BL/6J background (the latter provided by Caetano Reis e Sousa at the Francis Crick Institute, London, UK); CBA/Ca; and Balb/c mice were bred and maintained at the Francis Crick Institute under specific pathogen–free conditions. All protocols for breeding and experiments with animals were approved by the Home Office, UK, under project license P9C468066, and performed in accordance with the Animals (Scientific Procedures) Act 1986.

### Viruses

For in vivo infections, A/Puerto Rico/8/1934 (PR8, H1N1), a reassortant between PR8 and A/Hong Kong/68 (X31, H3N2), and A/California/04/09 (Cal/09, H1N1; kind gifts from J. Skehel and J. McCauley, Francis Crick Institute, London, UK) were grown in the allantoic cavity of 10-d-embryonated hens’ eggs and were free of bacterial, mycoplasma, and endotoxin contamination. For in vitro stimulation, viruses were grown in Madin-Darby canine kidney (MDCK) cells and tested negative for bacterial, mycoplasma, and endotoxin contamination. All viruses were stored at −80°C and titrated on MDCK cells. Virus was quantified in infected lungs by quantitative RT-PCR (RT-qPCR) for the influenza *matrix* gene on cDNA from whole lungs, normalized to the housekeeping gene *Hprt* or titrated on MDCK cells.

### Infections

Mice (6–12 wk old) were infected intranasally with influenza virus as indicated, in 30 µl PBS under light anesthesia (3% isoflurane). Pre-infection body weights were recorded, and mice were weighed daily (at similar times of day) and monitored for clinical symptoms. Mice reached humane endpoints after the loss of 20% of their initial starting weight or at a clinical score of >5. Clinical scores were determined by piloerection, hunched posture, partially closed eyes, labored breathing, hypothermia, decreased movement, movement only on provocation (1 point each), and absence of movement on provocation (2 points).

### Treatments

Fully phosphorothioated oligodeoxynucleotides were synthesized by TIB MOLBIOL (or Innaxon where indicated) and administered intranasally (90 µg in 30 µl PBS) under light anesthesia (3% isoflurane) on day 4 after infection. To deplete pDCs, mice were treated with anti–PDCA-1 monoclonal antibody, clone 927 (Bio X Cell; BE0311, RRID:AB_2736991), anti–Siglec-H monoclonal antibody, (Bio X Cell; BE 0202, RRID:AB_10949014), or isotype control (IgG2b; Bio X Cell; BE 0086, RRID:AB_1107791) 24 h before and after infection (500 µg per 200 µl i.p.) and every 48 h thereafter (200 µg per 200 µl i.p.). Mice were treated with anti-Ly6G monoclonal antibody, clone 1A8 (Bio X Cell; BE0075-1, RRID:AB_1107721) or IgG2b isotype-matched control on days 5 and 6 (300 µg per 200 µl i.p.) and 8 and 9 (150 µg per 200 µl i.p.) after infection to deplete neutrophils. For monocyte depletion, mice were treated i.p. with 20 μg anti-CCR2 in 200 µl PBS (MC-21, RRID:AB_2314128, provided by M. Mack) or isotype control for 3 d consecutively from day 5 after infection.

### BAL

On day 6 after infection, mice were euthanized (600 mg/kg pentobarbital and 16 mg/kg mepivacaine), and BALF was collected through catheter insertion intratracheally, injecting and retracting 400–500 µl of PBS. BALF was centrifuged at 350 *g* for 5 min at 4°C, and supernatants were stored at −80°C. Pelleted cells in the BALF were counted and analyzed by flow cytometry.

### In vitro stimulation of murine pDCs

Bone marrow cells were obtained by mechanical flushing of femurs and tibias. Red blood cells were lysed using ammonium chloride, and cells were cultured in Flt3-ligand (PeproTech; 250-31L; 150 ng/ml) supplemented culture media (10% fetal calf serum [PAA], L-glutamine, penicillin, streptomycin, and β-mercaptoethanol in RPMI-1640). Medium was replenished on day 4 of culture, and cells were harvested on day 8. Harvested cells were preincubated with Fc block and biotin-conjugated B220 (BioLegend; 103204, RRID:AB_312989) before 30-min incubation with anti-biotin–conjugated magnetic beads (Miltenyi Biotec; 130-048-101). pDCs were then positively selected using LS Columns (Miltenyi Biotec; 130-042-401) and the QuadroMACS separator (Miltenyi Biotec; 130-091-051), as per manufacturer’s instructions. pDCs, found to be 69.3 ± 3.58% pure based on FSC^low^, SSC^low^, PDCA-1^+^, Siglec-H^+^, B220^+^, CD11b^−^, CD11c^int^ as analyzed by flow cytometry, were seeded at 2 × 10^5^ cells per well in 200 µl in a 96-well plate and rested for 24 h before stimulation with X31 (multiplicity of infection [MOI] as indicated), ssRNA polyU (1 µg/ml; InvivoGen; tlrl-sspu) complexed with LyoVec (InvivoGen; lyec-2), or CpG-A 2216 (6 µg/ml, InvivoGen; tlrl-2216-5) in the presence or absence of IRS661 or Crtl ODN (1 µM).

### In vitro stimulation of human pDCs

Frozen human peripheral blood BDCA-4^+^ cells were bought directly from Stem Cell Technologies (70038). Cells were obtained in the U.S. in compliance with applicable federal, state, and local laws, regulations, and guidance. Cells were obtained from donors who were voluntarily participating in a donor program approved by an institutional review board, the Food and Drug Administration, or an equivalent regulatory authority and have waived any rights generated from the research applications. The informed consent obtained by the vendor covers the research performed here. After thawing and washing according to manufacturer’s instructions, the cells were seeded at 5 × 10^4^ cells per well in 100 µl in a 96-well plate and rested for 2 h before stimulation with X31 (MOI 1) in the presence or absence of IRS661 (1 µM).

### In vitro stimulation of murine monocytes

Bone marrow cells were obtained by mechanical flushing of femurs and tibias. Monocytes were directly isolated by negative selection using the monocyte isolation kit according to manufacturer’s instructions (Miltenyi Biotec; 130-100-629) and had a purity of >95%. Monocytes were seeded at 2 × 10^5^ cells per well in 200 µl in a 96-well plate and rested for 2 h before stimulation with X31 (MOI 1) in the presence or absence of IRS661 (1 µM).

### Quantification of cytokines

IL-6 was measured using ELISA mouse Ready-set-Go kit (Thermo Fisher Scientific; 88-7064-22, RRID:AB_2574986) as per the manufacturer’s instructions and read on a Safire II plate reader (Tecan). 2-Plex ProcartaPlex (Thermo Fisher Scientific; EPX02A-22187-901) was used to measure the concentrations of IFN-α/β, and the ProcartaPlex Cytokine and Chemokine Mouse 36-Plex (Thermo Fisher Scientific; EPXR360-26092-901) was used to assess the concentrations of 36 cytokines (IFN-γ, IL-1β, IL-2, IL-4, IL-5, IL-6, IL-12p70, IL-13, IL-18, TNFα, IL-9, IL-10, IL-17A [CTLA-8], IL-22, IL-23, IL-27, G-CSF [CSF-3], IFN-α, IL-3, IL-15/IL-15R, IL-28, IL-31, IL-1α, LIF, ENA-78 [CXCL5], M-CSF, Eotaxin [CCL11], GROα [CXCL1], IP-10 [CXCL10], MCP-1 [CCL2], MCP-3 [CCL7], Mip-1α [CCL3], Mip-1β [CCL4], Mip-2, and RANTES [CCL5]), read on a Luminex 100 (Bio-Rad; RRID:SCR_018025).

### RNA isolation and RT-qPCR

Lungs were harvested and stored in RNAlater (Ambion; AM7021) at −80°C. Lungs were homogenized with a Kinematica Polytron PT 10-35 homogenizer in 3 ml RNeasy Lysis Buffer (RLT; Qiagen; 79216) plus β-mercaptoethanol. pDCs and cells from the BAL were lysed in 350 µl RLT plus β-mercaptoethanol. RNA was isolated using the Qiagen RNeasy mini kit (Qiagen; 74106), according to the manufacturer’s instructions. 500 ng total RNA was reverse transcribed using the qPCRBIO cDNA synthesis kit (PCRBiosystem; PB30.11-10) as per manufacturer’s instructions. RT-qPCR was performed on an Applied Biosystems Quantstudio 3 real time PCR machine with qPCRBIO Probe Mix Lo-ROX (PCRBiosystem; PB20.21-51), and TaqMan primers. Results were normalized to the housekeeping gene *Hprt*. The following probes (Applied Biosystems; 4331182) were used: *Hprt1* (Mm00446968_m1), *Il6* (mm00446190_m1), *Ifna4* (Mm00833969_s1), *Ifnb1* (mm0439552_s1), *TNFa* (mm00443258_m1), *HPRT1* (human, Hs00369813_m1), *IFNA4* (human, Hs00855471_g1), *IFNB1* (human, Hs01077958_s1), *IL6* (human, Hs00985639_m1), and *TNFA* (human, Hs01113624_g1). Primers for influenza virus *matrix* gene were as follows: forward: 5′-AAG​ACC​AAT​CCT​GTC​ACC​TCT​GA-3′; reverse: 5′-CAA​AGC​GTC​TAC​GCT​GCA​GTC​C-3′; and probe: 5′-TTT​GTG​TTC​ACG​CTC​ACC​GT-3′.

### Flow cytometry

Cells from BAL were preincubated with anti-FcγRIII/II (Fc block) in FACS buffer before a 30-min incubation at 4°C with the following fluorochrome-labeled antibodies: anti-B220 APC (clone RA3-6B2, dilution 1:200, BioLegend; 103212, RRID:AB_312997), anti-B220 PE-Cy7 (clone RA3-6B2, dilution 1:200, BioLegend; 103222, RRID:AB_313005), anti-CD3 APC (clone 17A2, dilution 1:100, BioLegend; 100236, RRID:AB_2561456), anti-CD3 FITC (clone 17A2, dilution 1:100, Tonbo Biosciences; 35–0032, RRID:AB_2621660), anti-CD4 BV605 (clone RM4-5, dilution 1:400, BioLegend; 100548, RRID:AB_2563054), anti-CD8 BV510 (clone 53-6.7, dilution 1:400, BioLegend; 100752, RRID:AB_2563057), anti-CD11b BV421 (clone M1/70, dilution 1:200, BioLegend; 101236, RRID:AB_11203704), anti-CD11c BV605 (clone N418, dilution 1:400, BioLegend; 117333, RRID:AB_11204262), anti-CD19 APC (clone 6D5, dilution 1:100, BioLegend; 115512, RRID:AB_313647), anti-CD19 APC-Cy7 (clone 1D3, dilution 1:200, BD Biosciences; 557655, RRID:AB_396770), anti-CD44 APC (clone IM7, dilution 1:200, eBioscience; 17-0441-83, RRID:AB_469391), anti-CD64 PE-Cy7 (clone X54-5/7.1, dilution 1:100, BioLegend; 139314, RRID:AB_2563904), anti-CD69 BV421 (clone H1.2F3, dilution 1:400, BioLegend; 104523, RRID:AB_2260064), anti-Ly6C BV785 (clone HK1.4, dilution 1:600, BioLegend; 128041, RRID:AB_2565852), anti-Ly6G FITC (clone 1A8, dilution 1:200, BioLegend; 127606, RRID:AB_1236494), anti-NKp46 APC (clone 29A1.4, dilution 1:100, BioLegend; 137608, RRID:AB_10612758), anti–Siglec-F APC-Cy7 (clone E50-2440, dilution 1:600, BD Biosciences; 565527, RRID:AB_2732831), anti–Siglec-H PE (clone 551, dilution 1:200, BioLegend; 129606, RRID:AB_2189147), anti-MHCII BV711 (clone M5/114.15.2, dilution 1:800, BioLegend; 107643, RRID:AB_2565976), anti-MHCII PE-Cy7 (clone M5/114.15.2, dilution 1:1,000, BioLegend; 107630, RRID:AB_2069376), anti–PDCA-1 BV650 (clone 927, dilution 1:400, BioLegend; 127019, RRID:AB_2562477), anti–PDCA-1 FITC (clone 927, dilution 1:2,000, BioLegend; 127008, RRID:AB_2028462), Fixable blue dead stain UV395 (1:400, Thermo Fisher Scientific; L23105), and Pro5 MHC H-2Db/Influenza A NP (ASNENMETM) Pentamer PE (1:10, ProImmune; F119-2B-G). Cells were then washed, fixed with 4% paraformaldehyde (PFA), analyzed on a BD LSRFortessa cell analyzer (BD Bioscience), and interpreted using the software FlowJo v10.6.2 (FlowJo, RRID:SCR_008520).

### Primary mouse and human tracheal epithelial cell culture

Isolation and culture of primary mouse tracheal epithelial cells were performed as previously described (You et al., 2002). Briefly, cells isolated by enzymatic treatment were seeded onto 0.4-µm-pore-size clear polyester membrane (Greiner) coated with a collagen solution. Cells were grown in submersion until confluent and then exposed to air to establish an air–liquid interface. Primary human bronchial epithelial cells were purchased from Lonza, which sources these cells ethically in the U.S. and in compliance with applicable federal, state, and local laws, regulations, and guidance, and upon approval by an institutional review board, the Food and Drug Administration, or equivalent regulatory authority. The informed consent obtained by the vendor covers the research performed here. Primary human bronchial epithelial cells were cultured as per manufacturer’s instructions. In brief, cells were expanded in a T-75 flask to 60% confluence and then harvested for seeding onto Transwells (Greiner) at 3 × 10^4^ cells per insert. At confluence, liquid was removed from the upper chamber to establish air–liquid interface.

### Viral infection of tracheal epithelial cell cultures

The apical surface of cultures was washed extensively to remove accumulated mucus before inoculation with influenza A virus. After incubation at 37°C for 1 h, the virus inoculum was removed, and the cultures were incubated in complete growth medium for 24 h. Aliquots of the supernatants were collected at different time points and titrated by ELISA as described. Cells were then lysed to extract RNA.

### Histology and histopathologic analysis

Whole lungs were inflated with intratracheal injection of 4% PFA, fixed overnight, also in 4% PFA, and then embedded in paraffin wax and sectioned at 4 µm. Sections were stained with H&E and then subjected to microscopic evaluation.

Histopathologic examination of lungs was performed by a board-certified veterinary pathologist (S.L. Priestnall). Sections from all lung lobes were examined, and in each case histopathologic scores were based on the regions with the most severe changes. A semiquantitative scoring system was used (0, normal; 1, minimal; 2, mild; 3, moderate; and 4, marked changes) for each parameter. Parameters assessed were bronchiolar damage (cilia loss, erosion), bronchiolar repair (epithelial hyperplasia), peribronchiolar neutrophils, and perivascular neutrophils. The total histopathology score for inflammation was generated as the mean of the separate inflammatory scores.

### Statistical analysis

All results are expressed as mean ± SD (apart from the weight loss, where it is mean ± SEM). Statistical significance was determined as P < 0.05, applying two-way ANOVA with appropriate multiple corrections test as necessary (when weight loss curves and when two genotypes and different treatments were compared), one-way ANOVA (when different treatments in one genotype were compared), or unpaired *t* test (when two treatments in one genotype were compared). For each figure, the statistical test used is mentioned in the figure legend. Survival curves were analyzed using log-rank test. All statistics were performed with Prism 9 software (GraphPad, RRID:SCR_002798). In general, sample sizes were chosen to adhere to the UK Home Office 3R principles, while providing appropriate statistical power; thereby all in vivo experiments use at least three mice, and each symbol in graphs represents one animal. In vitro experiments are performed in triplicate, with samples from at least two individual mice. Symbols indicating significance are as follows: *, P < 0.05; **, P < 0.01; ***, P < 0.001; and ****, P < 0.0001. P values at the bottom of weight loss or survival curves indicate the difference to IgG2b- or PBS-treated control group as indicated.

### Online supplemental material

Fig. S1 shows additional experiments demonstrating activating function of the control ODN 1 and an alternative control ODN 2. Fig. S2 shows the experimental setup, optimal route of application of IRS661, flow cytometry gating strategy, measurements of viral load, additional host and virus strain combinations, and data on adaptive immunity. Fig. S3 shows further characterization of the CS in IFNAR1 KO mice, including data on viral load, αPDCA-1–mediated cell depletion, and cytokine levels.
